# CT and MR imaging features of soft tissue rhabdoid tumor: compared with rhabdomyosarcoma in children

**DOI:** 10.3389/fped.2023.1199444

**Published:** 2023-07-21

**Authors:** Jing Sheng, Ting-Ting Li, Huan-Huan Zhang, Hua-Feng Xu, Xue-Mei Cai, Rong Xu, Qiong-Qiong Ji, Yu-Meng Wu, Ting Huang, Xiu-Jun Yang

**Affiliations:** Department of Radiology, Shanghai Children's Hospital, School of Medicine, Shanghai Jiao Tong University, Shanghai, China

**Keywords:** computed tomography scan (CT scan), magnetic resonance imaging, rhabdoid tumor, rhabdomyosarcoma, differential diagnosis

## Abstract

**Objective:**

To assess the computed tomography (CT) and magnetic resonance (MR) imaging characteristics of soft tissue rhabdoid tumors (RT) and compare them with those of rhabdomyosarcoma (RMS).

**Methods:**

We conducted a retrospective analysis of 49 pediatric patients from 2011 to 2022, comprising 16 patients with soft tissue RT and 33 patients with RMS who underwent CT or MRI scans. Key imaging features, as well as clinical and pathological data, were compared between the two groups. The multivariate logistic regression analysis was used to determine independent differential factors for distinguishing soft tissue RT from RMS, and the model was established. The final prediction model was visualized by nomograms and verified internally by using a bootstrapped resample 1,000 times. The diagnostic accuracy of the combined model was assessed in terms of discrimination, calibration, and clinical utility.

**Results:**

Age, sex, number of lesions, and primary locations were similar in both groups. The imaging characteristics, including margin, calcification, surrounding blood vessels, and rim enhancement, were associated with the two groups of soft tissue tumors, as determined by univariate analysis (all *p *< 0.05). On multivariate logistic regression analysis, the presence of unclear margin (*p*-value, adjusted odds ratio [95% confidence interval]: 0.03, 7.96 [1.23, 51.67]) and calcification (0.012, 30.37 [2.09, 440.70]) were independent differential factors for predicting soft tissue RT over RMS. The presence of rim enhancement (0.007, 0.05 [0.01, 0.43]) was an independent differential factor for predicting RMS over soft tissue RT. The comprehensive model established by logistic regression analysis showed an AUC of 0.872 with 81.8% specificity and 81.3% sensitivity. The decision curve analysis (DCA) curve displayed that the model achieved a better net clinical benefit.

**Conclusion:**

Our study revealed that the image features of calcification, indistinct margins, and a lack of rim enhancement on CT and MRI might be reliable to distinguish soft tissue RT from RMS.

## 1. Introduction

Rhabdoid tumor (RT) is a rare, highly aggressive, and fast-progressing type of sarcoma with a high mortality rate. It is histologically characterized by rhabdoid cells and genetically characterized by the loss of function of the chromatin remodeling complex SWI/SNF induced by gene deficiency of *SMARCB1* (also known as *hSNF5, INI1, and BAF47*) on chromosome 22q11.23 or *SMARCA4* (also known as *BRG1* and *Hsnf2b*) on chromosome 19p13.2 (1, 2). RT was initially described by Beckwith et al. in 1978 in young children with primary renal tumors, but it can occur in various locations and at any age (3). The most common sites are the central nervous system (also known as the atypical teratoid/rhabdoid tumor, AT/RT) and the kidneys (4). Unlike previous studies, the rhabdomyosarcoma (RMS) and soft tissue RT mentioned in this article exclude those that originate in internal organs or the central nervous system.

The annual incidence of soft tissue RT in children older than one year is less than 0.1% per million (5). It is frequently mistaken with more common soft tissue sarcomas, such as RMS, which likewise primarily affects young children. In the United States, around 4.5 per million children and adolescents under the age of 20 are diagnosed with RMS every year (6). RMS and soft tissue RT may be difficult to differentiate since patients with these disease entities typically present with nonspecific clinical symptoms, such as pain or a palpable lump, and rhabdoid differentiation with distinctive nuclear pleomorphism and atypia histologically (7). Despite these similarities, soft tissue RT and RMS have widely differing prognoses. According to a study by the International Society of Pediatric Oncology (SIOP), the 5-year overall survival (OS) rate for RMS can approach 74% (8). However, soft tissue RT has a poor prognosis (9). Madigan et al. reported a 37.5% 2-year OS and 5-month median survival for 8 patients with soft tissue RT (10). A median survival of less than one year for soft tissue RT was also discovered in another study (4). Thus, it is essential to differentiate early soft tissue RT from RMS.

Imaging as a noninvasive method plays a vital role in the initial diagnosis. Unfortunately, the imaging characteristics of soft tissue RT is yet to determined (11). To the best of our knowledge, this is the largest retrospective single institutional imaging study primarily focused on comparing soft tissue rhabdoid tumors to rhabdomyosarcomas. In this study, we analyzed the clinicopathologic and imaging characteristics of 16 patients diagnosed with RT by surgical pathology between January 2011 and December 2022 in order to improve early CT and MRI diagnostic accuracy and reduce the rate of misdiagnosis of this aggressive tumor, and compared with RMS.

## 2. Materials and methods

### 2.1. Patients

This case-control study was approved by the Ethical Committee of Children's Hospital of Shanghai/Shanghai Children's Hospital, Shanghai Jiao Tong University (Approval number: 2022R160-E01), and informed consent was waived due to the retrospective nature of the analyses and the use of anonymized medical records.

**Figure 1:**
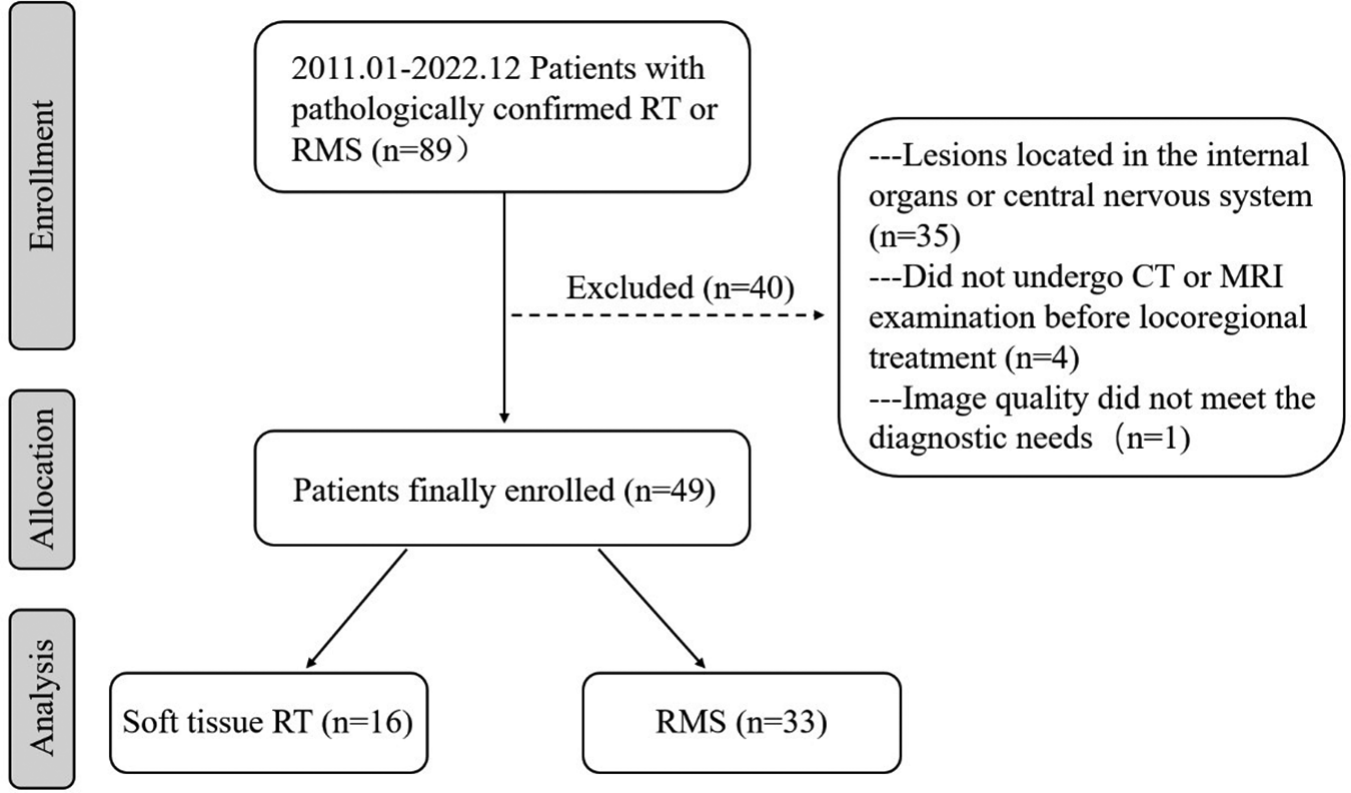
Flow chart visualizing the patient selection process for the study.

From January 2011 to December 2022, 89 individuals with soft tissue RT or RMS were identified after a search of the medical files and the picture archiving and communication systems (PACS) at our institution (Figure 1). The inclusion criteria for this study were as follows: (a) confirmation of soft tissue RT or RMS by surgical pathology; (b) multidetector CT and/or MRI examination was performed within 6 months before surgery and chemotherapy. Exclusion criteria were as follows: (a) lesions located in the internal organs or central nervous system; (b) patients did not undergo CT or MRI examination before locoregional treatment; (c) image quality did not meet the diagnostic needs. Ultimately, 49 patients with soft tissue tumors, comprising 33 patients with RMS and 16 patients with soft tissue RT, were enrolled in this study. Data were collected on patient demographics, clinical symptoms, and immunohistochemistry.

### 2.2. Imaging protocols for CT and MRI

Among the 49 patients, 45 underwent multi-slice spiral CT scans, 19 underwent MRI scans, and 15 underwent both CT and MRI scans. All of the patients fasted for four hours to before the scan, and those who couldn't cooperate with the CT or MRI tests were given 10% chloral hydrate (0.5 ml/kg) orally for sedation prior to the test.

The CT examinations were performed using 64-multidetector CT scans (Lightspeed Ultra 64, GE Medical Systems, USA or Aquilion Ultra 64, TOSHIB, Japan), with the following parameters: a layer thickness of 0.625 mm, interval of 2.5 mm, pitch of 0.984, tube voltage of 100 kV, and tube current of 240 mA. Coronal and transverse multiplanar reconstruction CT images were reconstructed with 3 mm section thickness. After the acquisition of unenhanced CT, 2 ml/kg of Iohexol (350 mg/ml, Schering, Germany) was administered intravenously at a rate of 1.8–2.5 ml/s using an automatic power injector (OptiVantage DH; Mallinckrodt, St. Louis, Mo) into a 22- or 24-gauge intravenous cannula, without normal saline bolus administration. Trip-phase enhanced images were obtained at 15–20 s, 40–50 s, and 120–180 s after intravenous contrast injection.

All patients used a 3.0 T scanner (Ingenia, Philips Medical Systems, Best, the Netherlands). To achieve optimal resolution, coils were selected according to the respective body region examined. Scanning methods: conventional axial, coronal, and sagittal scans, including T1-weighted images (T1WI), T2-weighted images (T2WI) and fat-suppressed T2-weighted images (Fs-T2WI), with a slice thickness of 5 mm and an interval of 4 mm. A single-shot echo sequence was used to generate diffusion-weighted images (DWIs). The diffusion gradients were applied in three orthogonal directions (x, y, and z). After 0.2 ml/kg Gd-DTPA (3 ml/s) was injected intravenously, sagittal, axial, and coronal T1WI pictures were acquired. Detailed scan parameters are shown in Table 1.

**Table 1:** MRI sequences and parameters.

Sequence	T1WI	T1WI	T2WI	T2WI	T2WI	DWI	DWI	CE-T1WI	CE-T1WI
Plane	Axial	Sagittal	Axial	Axial	Sagittal	Axial	Sagittal	Axial	Sagittal
TR (ms)	615	600	2,800	2,800	3,000	2,255	2,255	580	590
TE (ms)	18	15	85	80	80	65	65	15	16
Thickness/gap (mm)	4/0.4	4/0.4	4/0.4	4/0.4	3/0.3	4/0.4	4/0.4	4/0.4	3/0.3
Matrix (mm)	300 × 200	300 × 200	368 × 210	368 × 210	368 × 210	88 × 100	88 × 100	300 × 200	300 × 200
FOV (cm)	24 × 20	24 × 20	24 × 20	24 × 20	22 × 20	24 × 20	22 × 20	24 × 20	22 × 20
Fat suppression	No	No	No	Yes	No	Yes	Yes	No	Yes

T1WI, T1-weighted imaging; T2WI, T2-weighted imaging; DWI, diffusion-weighted imaging; CE, contrast-enhanced; TE, echo time; TR, repetition time; FOV, field of view.

### 2.3. Imaging analysis

The CT and MRI pictures were assessed by two radiologists with more than ten years of expertise who were blinded to the ultimate diagnosis. Individual interpretation differences were settled through consensus. The following imaging characteristics were noted and documented: (a) number (single or multiple); (b) location (head and neck/trunk/extremities); (c) size (maximum tumor diameter); (d) morphology (multilobulated/round/others); (e) margin (clear or unclear); (f) density/signal intensity (lower or higher than normal muscle/cystic necrosis/calcification/hemorrhage/fat); (g) peripheral cyst (present or absent); (h) surrounding blood vessels (present or absent); (i) rim enhancement (present or absent). Other radiologic features, such as adjacent bone destruction and metastatic sites, were also evaluated. The largest tumor diameter was measured in any axial, coronal, or sagittal plane on both CT and MRI. An unclear margin was defined as lacking clear borders between the tumor and surrounding structures on unenhanced images.

### 2.4. ADC histogram analysis

DWI examinations were performed on three cases of soft tissue RT and thirteen cases of RMS. The ADC maps were automatically created for the obtained images with the software built into the MR unit using b values of 0 and 1,000 s/mm^2^. All MRI data were then transferred to an offline workstation in DCM format. Two experienced pediatric radiologists, with 10 and 20 years of MRI experience, independently assessed the MRI images and identified the regions of interest (ROI), which included the entire tumor, for histogram analysis using MaZda software (version 4.6, The Technical University of Lodz, Institute of Electronics, http://www.eletel.p.lodz.pl/mazda/). The software automatically extracted nine histogram parameters, such as the mean, variance, skewness, and kurtosis, as well as the 1st (ADCp1), 10th (ADCp10), 50th (ADCp50), 90th (ADCp90), and 99th (ADCp99) percentiles of ADC.

### 2.5. Statistics analysis

Statistical analyses were conducted using SPSS Statistics version 25 (IBM) and R version 4.2.2 (http://www.r-project.org) statistical software. The Shapiro-Wilk test was used to determine data normality, and Levene's test was used for homogeneity of variance. The mean and standard deviation (SD) were used to describe continuous variables with a normal distribution, and compared with the student *t*-test. The median and interquartile ranges (IQRs) were used to describe continuous variables with a non-normal distribution, and compared with the Mann-Whitney *U*-test. The chi-square or Fisher's exact test was used to compare categorical variables, and the results are expressed as numbers and percentages of patients. The variables of imaging with *p *< 0.05 in univariate analysis were included in the multivariate logistic regression analysis (forward LR) to determine the independent factors for distinguishing soft tissue RT and RMS. For each risk factor, odds ratios (OR) were calculated as relative risk estimates with 95% confidence intervals (CI). Additionally, Cohen's kappa was used to calculate the inter-observer agreement between the two radiologists. A two-tailed *p *< 0.05 was considered to be statistically significant.

### 2.6. Model development and validation, and evaluation

Combining independent predictors identified by multivariate logistic regression (forward LR) to form a model for discriminating between soft tissue RT and RMS. To verify the accuracy of the model, bootstrapping resampling was performed 1,000 times, and the results were visualized using nomograms. The sensitivity, specificity, and area under the receiver operating characteristic (ROC) curve (AUC) were used to evaluate the model's discrimination. Calibration curve analysis was used to assess the consistency between the tumor types identified by the model and the actual tumor types. Additionally, decision curve analysis (DCA) was conducted to determine the clinical utility of the model by assessing the net benefits at various threshold probabilities. Kaplan–Meier (K–M) survival curves with the log-rank test were generated to evaluate the differences in survival rate between the two groups.

## 3. Results

### 3.1. Clinical and immunohistochemical findings

The clinical and immunohistochemical findings of soft tissue RT (*n* = 16) and RMS (*n* = 33) are presented in Table 2. There was no statistical difference in age (*p* = 0.518) or sex (*p* = 0.08) between the two groups. The incidence of limited motion of extremities in the soft tissue RT group was significantly higher than the RMS group (25% vs. 0%, *p* = 0.015). Immunohistochemically, the CK and EMA expression rates were significantly higher in soft tissue RT compared to RMS (93.3% vs. 13.8%, 92.9% vs. 15.0%, respectively, all *p* < 0.001). Of note, INI was expressed by all patients with RMS but not by those with soft tissue RT (100% vs. 0%, *p* < 0.001). Also, immunohistochemistry markers like MyoG, MyoD1, and DES were more often found to be positive in RMS patients than in soft tissue RT patients (all *p *< 0.001). The Ki-67 levels of soft tissue RT and RMS were 45.1 ± 28.7 and 57.8 ± 20.9, respectively (*p* = 0.095).

**Table 2:** Clinical and immunohistochemical characteristics of two groups.

Characteristic	Soft tissue RT (*n* = 16)	RMS (*n* = 33)	*p-*value
Age at onset (months)	27 (8,84)	37 (15,78)	0.518
Sex			0.08
Male	7/16 (43.8)	23/33 (69.7)	
Female	9/16 (56.3)	10/33 (30.3)	
Symptoms			
Pain	7/16 (43.8)	11/33 (33.3)	0.478
Limited motion of extremities	4/16 (25)	0/33 (0)	0.015
Palpable mass	11/16 (68.8)	29/33 (87.9)	0.219
Immunohistochemical expression			
INI1	0/15 (0)	22/22 (100)	<0.001
CK	14/15 (93.3)	4/29 (13.8)	<0.001
EMA	13/14 (92.9)	3/20 (15.0)	<0.001
MyoG	0/10 (0)	31/32 (96.9)	<0.001
MyoD1	0/9 (0)	27/32 (84.4)	<0.001
DES	2/11 (18.2)	30/32 (93.8)	<0.001
Ki-67	45.1 ± 28.7	57.8 ± 20.9	0.095

RT, rhabdoid tumor; RMS, rhabdomyosarcoma; INI1, integrase interactor 1; CK, cytokeratin; EMA, epithelial membrane antigen; MyoG, myogenin; MyoD1, myogenic differentiation 1; DES, desmin.

### 3.2. Imaging features and ADC histogram parameters

There were no statistically significant differences in terms of tumor number, location, size, or shape between the two groups. The peripheral cyst (50% vs. 24.2%, *p* = 0.071), hemorrhage (12.5% vs. 12.1%, *p* > 0.999), and fatty degeneration (0% vs. 6.1%, *p *> 0.999) also did not differ between the soft tissue RT and RMS. Unclear margin and calcifications were more commonly found in the soft tissue RT group than in the RMS group (87.5% vs. 57.6%, 43.8% vs. 12.1%, respectively, all *p *< 0.05). Surrounding blood vessels and rim enhancement were more frequently observed in patients with RMS compared with soft tissue RT (51.5% vs. 18.8%, 63.6% vs. 18.8%, respectively, all *p *< 0.05). As shown in Table 3.

**Table 3:** Comparison of imaging and ADC histogram parameters of two groups.

Parameter	Soft tissue RT (*n* = 16)	RMS (*n* = 33)	*p-*value
Number of lesions			>0.999
Single	15/16 (93.8)	30/33 (90.9)	
Multiple	1/16 (6.3)	3/33 (9.1)	
Location			0.242
Head and neck	5/16 (31.3)	14/33 (42.4)	
Trunk	11/16 (68.8)	15/33 (45.5)	
Extremities	0/16 (0)	4/33 (12.1)	
Maximum tumor diameter (cm)	8.3 ± 4.3	7.8 ± 6.3	0.797
Shape			>0.999
Multilobulated	2/16 (12.5)	3/33 (9.1)	
Round	5/16 (31.3)	12/33 (36.4)	
Others	9/16 (56.3)	18/33 (54.5)	
Unclear margin	14/16 (87.5)	19/33 (57.6)	0.036
Peripheral cyst	8/16 (50)	8/33 (24.2)	0.071
Calcification	7/16 (43.8)	4/33 (12.1)	0.034
Hemorrhage	2/16 (12.5)	4/33 (12.1)	>0.999
Fatty degeneration	0/16 (0)	2/33 (6.1)	>0.999
Surrounding blood vessels	3/16 (18.8)	17/33 (51.5)	0.029
Rim enhancement	3/16 (18.8)	21/33 (63.6)	0.003
Adjacent bone destruction	8/16 (50)	8/33 (24.2)	0.071
Attenuation on unenhanced CT			0.530
Iso or low	13/14 (92.9)	30/31 (96.8)	
High	1/14 (7.1)	1/31 (3.2)	
T1 signal intensity			0.517
Iso or low	6/6 (100)	10/13 (76.9)	
High	0/6 (0)	3/13 (23.1)	
T2 signal intensity			0.316
Iso or low	1/6 (16.7)	0/13 (0)	
High	5/6 (83.3)	13/13 (100)	
Lymph node enlargement	7/16 (43.8)	12/33 (36.4)	0.619
Recurrence	1/16 (6.3)	7/33 (21.2)	0.359
Number of metastases			0.637
Single metastases	4/7 (57.1)	4/10 (40)	
≥2 metastases	3/7 (42.9)	6/10 (60)	
ADC histogram parameters			
Mean	982.1 ± 371.9	928.3 ± 392.4	0.832
Variance	40,697.7 ± 9,583.6	24,416.9 ± 20,340.7	0.206
Skewness	0.3 ± 0.8	0.4 ± 0.4	0.678
Kurtosis	0.8 ± 1.9	0.6 ± 0.7	0.923
ADCp1	523.3 ± 351.7	647.9 ± 307.4	0.546
ADCp10	730.7 ± 308.6	767.5 ± 351.6	0.870
ADCp50	982.3 ± 417.7	920.1 ± 392.8	0.810
ADCp90	1,232.7 ± 375.2	1093.0 ± 440.2	0.621
ADCp99	1,468.0 ± 318.1	1287.0 ± 487.3	0.555

RT, rhabdoid tumor; RMS, rhabdomyosarcoma; CT, computed tomography; ADC, apparent diffusion coefficient.

**Figure 2:**
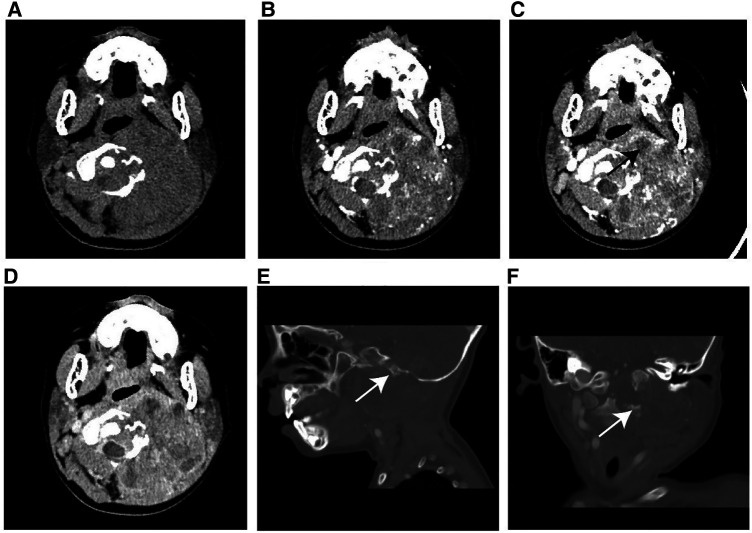
Typical instance of soft tissue RT in a female infant of 36 months (clinical manifestations: left neck swelling and pain for a month). (**A**) Non-enhanced CT scans revealed a moderately hypodense mass. (**B–D**) On multi-phase enhancement, the density of the tumor was higher than that of the adjacent muscle layer, with CT values for each period being around 56HU, 64HU, and 82HU, respectively. It showed heterogeneous enhancement and peripheral cyst (arrow). (**E,F**) Sagittal and coronal images revealed bone destruction in the adjacent skull base and multiple cervical vertebrae (arrow).

**Figure 3:**
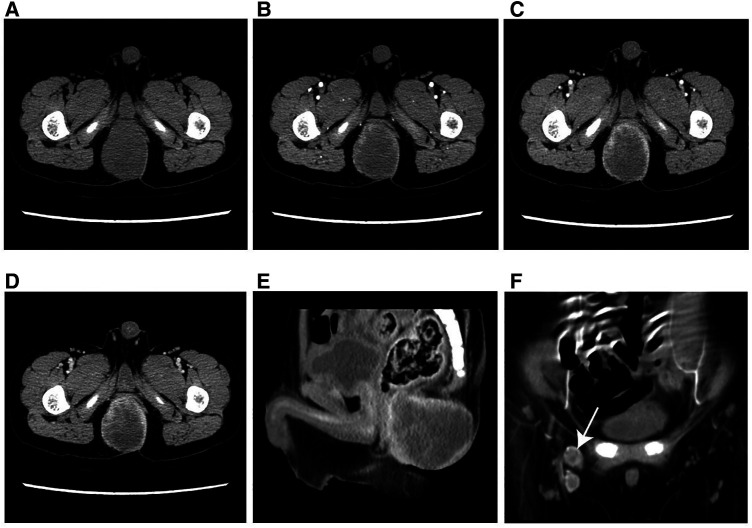
Typical example of RMS in a male infant aged 49 months (clinical manifestations: growing soft tisssue mass for 3 months). (**A**) The axial CT image revealed a well-defined, hypodense parenchymal mass measuring 47 mm in diameter. (**B–D**) On multi-phase enhancement, the CT values for each period were around 39 HU, 40 HU, and 60 HU, respectively, and displayed rim enhancement. (**E**) The sagittal CT image showed an indistinct boundary between the lesion and anterior rectum. (**F**) The coronal CT image presented enlarged lymph nodes in the right groin area (arrow).

**Figure 4:**
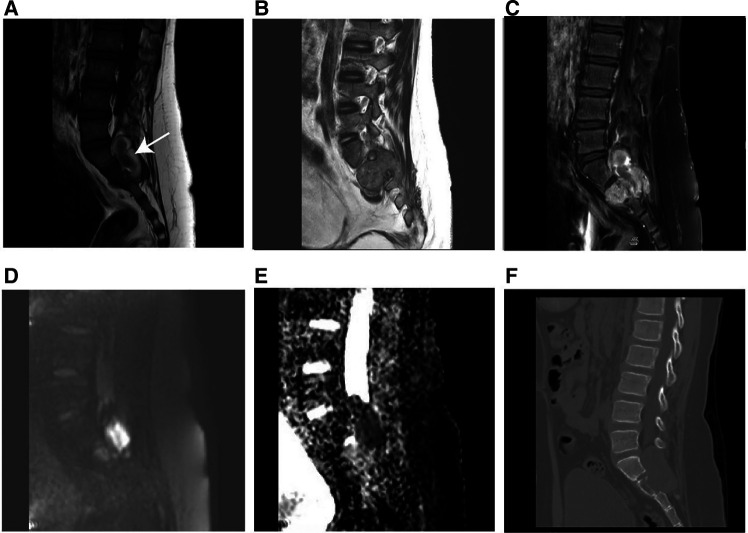
MR imaging results of soft tissue RT in a male of 90 months (clinical manifestations: sacrococcygeal paroxysmal pain for 1.5 years, gradually aggravated). (**A**) T1-weighted MR imaging indicated a moderately hypointense mass with hemorrhage (arrow). (**B**) T2­weighted MR imaging showed a moderately hyperintense mass extending from the intervertebral foramen to the posterior right pelvic wall. (**C**) T1-weighted enhanced MR imaging revealed heterogeneous enhancement. (**D**) Hyperintensity on the DWI image. (**E**) The mean apparent diffusion coefficient (ADC) value of the mass was 0.722 × 10^−3^ mm^2^/s. (**F**) The sagittal CT image revealed vertebral bone destruction.

**Figure 5:**
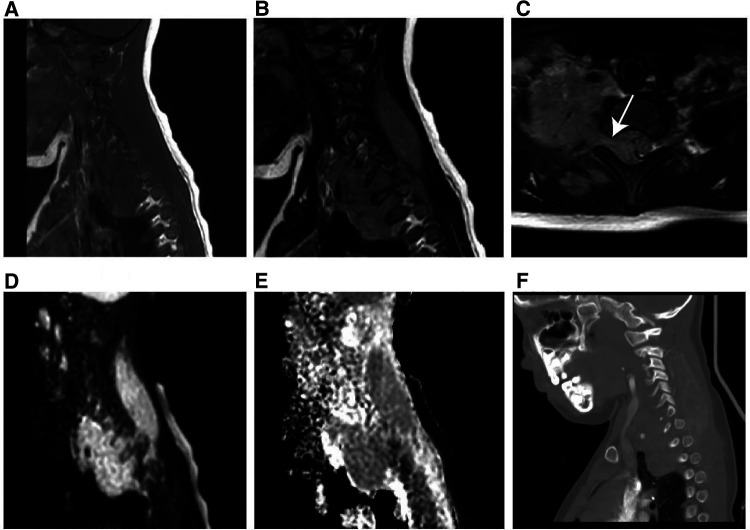
MR imaging results of a 60-month-old girl with RMS (clinical manifestations: unintentionally found a mass in the right neck for 20 days, accompanied by pain in the right upper limb for 15 days). (**A**) T1-weighted MR imaging revealed an isointense soft tissue mass. (**B**) T2-weighted MR imaging revealed a hyperintense mass. (**C**) Axial T2-weighted MR imaging revealed that the mass had expanded into the cone through the intervertebral foramen. (**D**) Hyperintensity on the DWI image. (**E**) The mean apparent diffusion coefficient value of the mass was 1.441 × 10^−3^ mm^2^/s. (**F**) The sagittal CT image revealed no obvious bone destruction.

On unenhanced CT images, thirteen patients with soft tissue RT exhibited iso- or hypodense relative to adjacent muscles (Figure 2), and only one exhibited hyperdense, which was not significantly different compared with RMS (Figure 3) (all *p *> 0.05). According to MRI characteristics, most cases in the two groups demonstrated Iso- or hypointense signal on T1-weighted MR images, and hyperintense signal on T2-weighted MR images (Figures 4, 5). Furthermore, none of ADC histogram parameters were statistically significant (all *p *> 0.05). Two radiologists evaluated subjective image indicators with an excellent degree of agreement (kappa values: 0.81–1).

### 3.3. Predictive value of imaging characteristics

In univariate analysis, four imaging features were significantly different between the two groups at a test level of *p *< 0.05 (Table 4). Based on multivariate logistic regression analysis (forward LR), an unclear margin (*p*-value, adjusted odds ratio [95% confidence interval]: 0.03, 7.96 [1.23, 51.67]), calcification (0.012, 30.37 [2.09, 440.70]) were independent differential factors for predicting soft tissue RT over RMS; rim enhancement (0.007, 0.05 [0.01, 0.43]) were independent differential factors for predicting RMS over soft tissue RT.

**Table 4:** Results of binary logistic regression analyses for differentiating soft tissue RT from RMS.

Variables	Univariate analysis		Multivariable analysis	
	OR (95% CI)	*p-*value	OR (95% CI)	*p-*value
Unclear margin	5.16 (1.01–26.45)	0.049	7.96 (1.23–51.67)	0.030
Calcification	5.64 (1.34–23.76)	0.018	30.374 (2.09–440.70)	0.012
Surrounding blood vessels	0.22 (0.05–0.91)	0.036		
Rim enhancement	0.13 (0.03–0.56)	0.006	0.047 (0.01–0.43)	0.007

RT, rhabdoid tumor; RMS, rhabdomyosarcoma; OR, odds ratio; CI, confidence interval.

### 3.4. Model development, evaluation and visualization

**Figure 6:**
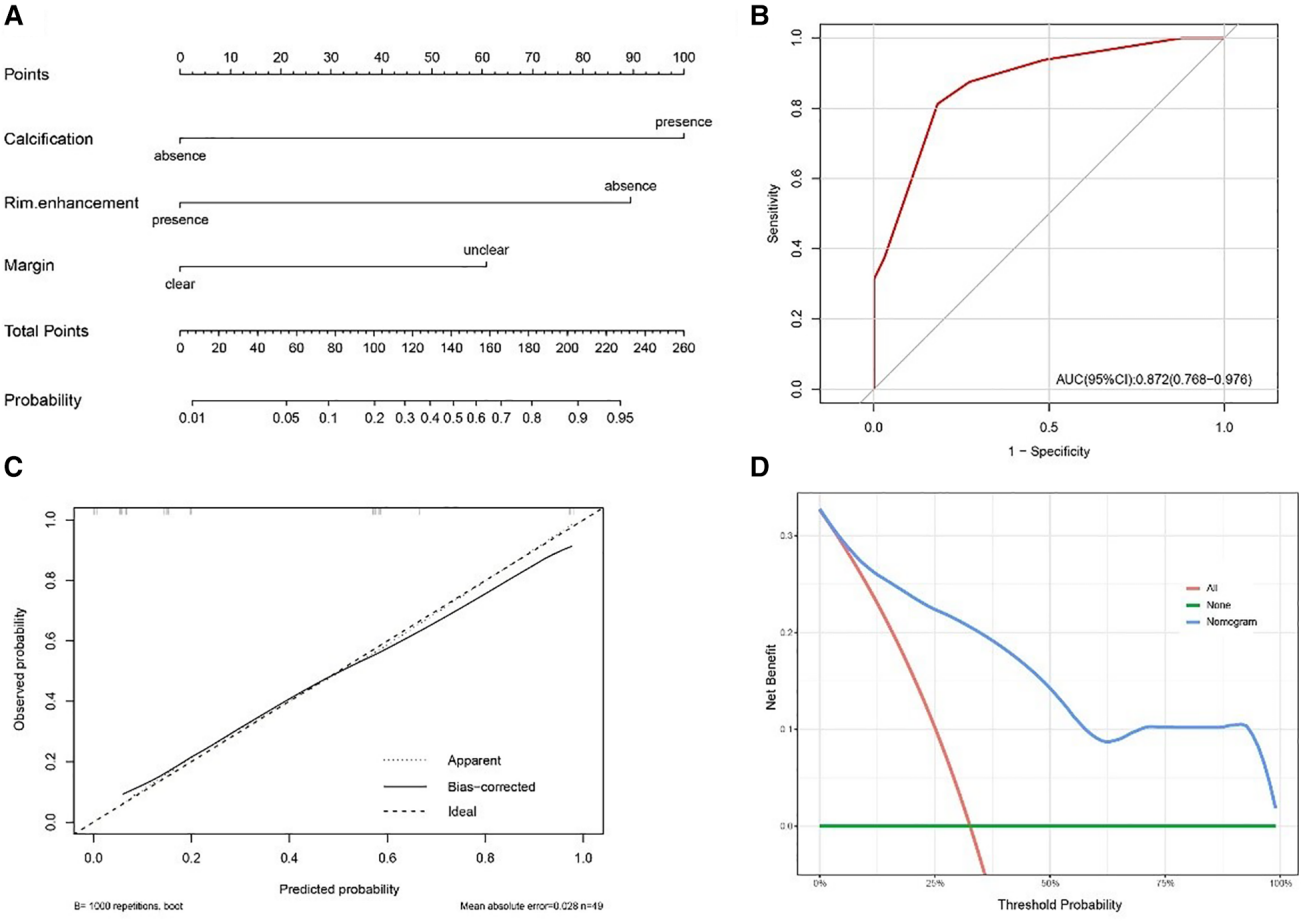
(**A**) Nomogram for distinguishing soft tissue RT from RMS. (**B**) The receiver operating characteristic curve (ROC) for the model. (**C**) The calibration plot for the model. (**D**) The decision curve analysis (DCA) for the model.

The nomogram was developed using the significant variables to make predictions about the occurrence of soft tissue RTs (Figure 6A). The results of further ROC curve analysis revealed that the area under the curve (AUC) was 0.872 with 81.8% specificity and 81.3% sensitivity, indicating excellent diagnostic performance (Figure 6B). Calibration curves (Figure 6C) demonstrated that the nomogram accurately estimated the agreement between observed outcome frequencies and predicted probabilities. Additionally, the DCA curve (Figure 6D) showed a high clinical net benefit over the “treat-all” or “treat-none” strategies and was of clinical application.

### 3.5. Follow-up

**Figure 7:**
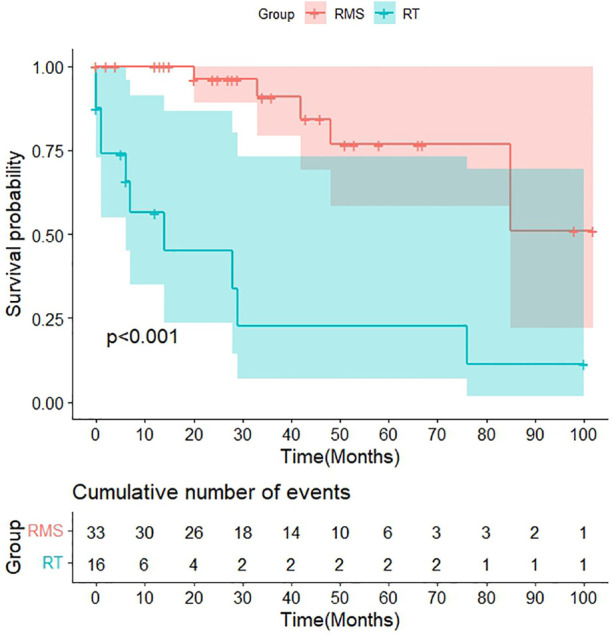
Survival curves of soft tissue RT and RMS.

The final follow-up date was December 2022, with a median follow-up time of 53 months. Clinical assessment and follow-up imaging revealed that recurrences occurred in 6.3% (1/16) of soft tissue RT patients and 21.2% (7/33) of RMS patients, with a mean time to recurrence of 19 vs. 16 months after diagnosis. Among the 43.8% (7/16) patients in the soft tissue RT group who had metastasis, 57.1% (4/7) had lung metastasis and 42.9% (3/7) had other metastatic locations, such as bone, lymph nodes, the omentum, etc. However, the bone (3/10) is the most common metastatic site for RMS patients, followed by the lung, lymph nodes, central nervous system, etc. The 3-year OS rate of the patients with RT were significantly lower than those of the patients with RMS (22.6% vs. 90.8%, *p *< 0.01). Furthermore, the median survival time for RT patients is only 14 months (Figure 7).

## 4. Discussion

Differentiating soft tissue RT from RMS is crucial for clinicians but difficult because there are many similar features between the two types. CT and MRI examinations, as noninvasive methods, have been widely used for preoperative diagnoses and evaluation of soft tissue tumors. Therefore, the present study aimed to identify reliable imaging factors that can best distinguish soft tissue RT from RMS through statistical analysis. The results of our study indicated that margin, calcification, and rim enhancement contributed significantly and independently to differential diagnosis between the two groups. Based on the three identified independent predictors, a multivariate logistic regression model was established, which showed high sensitivity (0.813) and specificity (0.818), indicating that these imaging features could achieve an excellent differential diagnostic performance for distinguishing soft tissue RT from RMS.

Univariate analysis showed the median age of patients with soft tissue RT was 27 months, and 56.3% (9/16) of patients were female, with the main clinical symptoms as palpable soft tissue masses (68.8%), which were consistent with previous reports (7, 12). Individuals with soft tissue RT are more likely than RMS patients to occur limited motion of extremities, possibly since soft tissue RT occurs frequently in deep axial locations, putting pressure on the spinal cord (13, 14). Our study found that 87.5% of RT tumors were in deep axial locations, while only 21.2% of RMS tumors were. Typically, RT cells were positive for epithelial markers and negative for muscle-derived markers, which is the exact opposite of the tumor of RMS (13, 15). Notably, the absence of the INI1 protein expression often used by pathologists to diagnose malignant rhabdoid tumors (16, 17). The WHO did not recommend using INI1 or a rare Brahma-related gene-1 (BRG1) mutation to diagnose AT/RT until 2016, and other types of RT are still waiting for clear regulations to adopt this method of diagnosis (18). Our pathological findings revealed that INI1 expression was absent in all patients except for one who was not tested, possibly because his symptoms appeared as early as 2011 when the INI1 gene was not seen as significant.

Owing to their rarity, radiological descriptions of soft tissue RTs in the current literature are few and non-specific, making identification challenging, despite several common imaging characteristics have been reported. Soft tissue RT mainly manifests as a large, heterogeneous, low-density mass that was hypo- to isointense to muscle on T1WI and hyperintense to muscle on T2WI, often with areas of necrosis (6, 19, 20). Cheng et al. reported that the median maximal diameter in 21 soft tissue RT patients was 7.4 cm (IQR: 4.8–11.8) (21). In our study, the median maximum diameters of the soft tissue RT group were 7.7 cm (IQR: 5.0–10.5), and more than 90% of patients showed iso- to hypodensity on unenhanced CT. Large or rapidly growing tumors are susceptible to tumor necrosis due to inadequate perfusion and oxygenation (22, 23). According to the results of our investigation, cystic necrosis was ineffective in distinguishing between soft tissue RT and RMS, probably because both had large tumor sizes that were not statistically different. Six soft tissue RT patients received MRI examinations, and the T1WI and T2WI signals were comparable to those previously reported.

Compared to RMS, soft tissue RT more frequently showed unclear margin in the study. It is likely due to the fact that RMS originates from skeletal muscle cells, which have a highly ordered arrangement forming a bundle-like structure with clear boundaries (24). However, soft tissue RT is a tumor of undetermined differentiation and invasive growth in the surrounding area (25). Furthermore, the study demonstrated that calcification was more prevalent in soft tissue RT than in RMS. Several studies reported that RT was associated with hypercalcemia, which may be due to RT secreting parathyroid hormone-related peptide (PTH-rP) and easily involving bones (26, 27). In addition, the rapid growth and metabolism of malignant RT cells, as well as the excessive proliferation of blood vessels in the tumor, make calcium salt deposition possible (28). Most studies agree with us that calcification and hemorrhage are uncommon in children with RMS (29, 30). As shown in the study, 63.6% (21/33) of RMSs and 18.8% (3/16) of soft tissue RTs displayed rim enhancement, which contributed significantly to the differential diagnosis between the two groups, and functioned as an independent predictor. RMS contains abundant fibrous tissue, and the contrast agent slowly permeates into the tumor center over time, resulting in noticeable rim enhancement during the initial stages of enhancement (31). Another reason may be that surrounding blood vessels are more common in RMS than RT.

Both RMS and soft tissue RT patients exhibit high DWI signals and low ADC values due to their high malignancy, rapid proliferation, and high cell density, which restrict the diffusion of water molecules. The present study found that the average ADC value for soft tissue RT and RMS was 0.98 × 10^−3^ mm^2^/s vs. 0.93 × 10^−3^ mm^2^/s, which is consistent with some studies reporting the RMS ADC value of (0.78–1.21) × 10^−3^ mm^2^/s (32, 33). We extracted nine distinguishing factors from the ADC graph using the gray histogram analysis method. The mean value reflects the average intensity of the image; the variance reflects the discrete degree of the grayscale distribution of the image; Skewness reflects the skew direction and degree of image gray distribution; The 1st, 10th, 50th, 90th, and 99th percentiles are used to describe values of observations below a given percentile, reflecting small variations within the image (34). However, no ADC value parameter was statistically significant between the two groups.

Our study has several limitations. First, the design of a single-center retrospective study might make selection bias inevitable. Second, the small sample size limited the statistical power of our results, even though this is the largest imaging study to date on soft tissue RT. As RMS and soft tissue RT are uncommon disorders in children, future multi-center research with sizable sample sizes will be required to confirm these findings.

## 5. Conclusion

The rarity of soft tissue RT frequently poses a diagnostic dilemma for radiologists.

Specifically, soft tissue RT is difficult to identify preoperatively from RMS. Compared to RMS, soft tissue RT had an ill-defined margin, calcification, and no rim enhancement, as determined by our study's radiological findings. The combination of above imaging characteristics could offer clinicians with additional preoperative imaging diagnoses and allow them to select the most effective treatment.

## Data Availability

The raw data supporting the conclusions of this article will be made available by the authors, without undue reservation.
